# Assessing the genetic profile of cytochrome P450 and glutathione S-transferases of patients diagnosed with acute myeloid leukemia

**DOI:** 10.1016/j.htct.2025.103759

**Published:** 2025-05-07

**Authors:** Gilmar de Andrade França, Luciana Nardinelli, Ricardo Rodrigues Giorgi, Thiago Pagliarini, Otávio César Carvalho Guimarães Baiocchi, Elvira Deolinda Rodrigues Pereira Velloso, Wellington Fernandes da Silva Jr, Eduardo Magalhães Rego, Israel Bendit

**Affiliations:** a Department of Hematology, Transfusion and Cell Therapy, University of Sao Paulo Medical School (HCFMUSP), Sao Paulo, SP, Brazil; b Laboratory of Medical Investigation in Pathogenesis and targeted therapy in Onco-Immuno-Hematology (LIM/31), Department of Hematology, Hospital das Clínicas HCFMUSP, Faculdade de Medicina, Universidade de Sao Paulo, Sao Paulo, SP, Brazil; c Department of Hematology, Cancer Institute of Sao Paulo, University of Sao Paulo Medical School (ICESP), Sao Paulo, SP, Brazil; d Hospital Alemão Oswaldo Cruz, Sao Paulo, SP, Brazil

**Keywords:** Pharmacogenomics, Acute myeloid leukemia, Polymorphism, Glutathione s-transferase, Cytochrome P450

## Abstract

**Objective:**

This study aimed to determine the frequency of genetic alterations as deletions and duplications in cytochrome P450 (*CYP450*) and glutathione S-transferases (*GST*) genes, as well as to investigate whether there is a relationship between these alterations and neutrophilic hematologic recovery in adult patients diagnosed with acute myeloid leukemia.

**Method:**

DNA samples from 70 patients diagnosed with acute myeloid leukemia were evaluated using the Multiplex Ligation-dependent Probe Amplification technique. The presence or absence of polymorphisms was compared regarding the time to neutrophilic recovery (neutrophil count ≥1.0 × 10^9^/L) using Kaplan-Meier curves, with the comparison between the curves being performed using the non-parametric log-rank test.

**Results:**

The median age of the participants was 57 years, with a higher proportion of females (57.2%) and white individuals (61.4%)’. A total of 76 polymorphisms (*CYP450* + *GST*) were identified, comprising 38 deletions and 38 duplications. Kaplan-Meier curves revealed that the neutrophilic recovery time was longer for the group with polymorphisms (p-value = 0.0056).

**Conclusion:**

The study demonstrated that *CYP450* and *GST* genes are polymorphic, and these polymorphisms may lead to longer neutrophilic recovery after induction treatment of acute myeloid leukemia remission.

## Introduction

The National Human Genome Research Institute (NHGRI) of the National Institutes of Health (NIH) of the United States of America defines pharmacogenomics as the area of medicine whose primary objective is to evaluate the relationship between the genetic profile and the variability in responses to drug therapy, and toxicities.^1^^,^^2^ According to information from the National Cancer Institute (INCA), 11,540 new cases of leukemia are expected in the country in 2024 (www.gov.br/inca/pt-br/assuntos/cancer/tipos/leucemia).^3^^,^^4^

Among all leukemias, acute myeloid leukemia (AML) is the most frequent in the adult population, characterized by the disordered growth of immature blood cells called myeloblasts ^5^^,^^6^. Various obstacles, like the need for early diagnosis, the biochemical characteristics of the disease itself, the emergency profile of starting treatment, and the difficulty of accessing new non-cytotoxic chemotherapy treatments, may influence therapeutic success.^7^^,^^8^ However, among these challenges, the genotypic variability of patients in respect to the metabolism of drugs used to treat AML stands out. This variability can contribute to increases in toxicity of medications and influence the clinical outcome.^9^^,^^10^

With advancing precision medicine and personalized medicine, more and more studies in pharmacogenomics are being conducted.^11^ These studies aim to unveil the genetic variability underlying the pharmacodynamics and pharmacokinetic response, which brings greater benefits and less harm to the patient.^12^ Regarding harm, both adverse drug reactions (ADR) and drug resistance mechanisms can be cited, which can lead to failures and relapses during treatment.^13^^,^^14^

Cytochrome P450 (CYP450) is a superfamily of enzymes related to various reactions in the body, from the biosynthesis of steroids and fatty acids to the biotransformation of exogenous substances.^15^

In the human genome, 57 *CYP450* genes that encode functional proteins have been found, and grouped according to their homology into 18 families and 44 subfamilies. A significant number of copy number variations have been noted in these genes, which may influence the biotransformation of drugs.^16^^,^^17^

The main genes belonging to the large cytochrome P450 family are: *CYP1A1, CYP1A2, CYP2A6, CYP3A4, CYP3A5, CYP1B1, CYP2B6, CYP2C9, CYP2C19, CYP2D6*, and *CYP2E1*.^18^

The genes that encode the glutathione S-transferases (GST) enzymes present considerable polymorphisms depending on ethnic variations and can suffer both homozygous gene deletions, leading to loss of function, and heterozygous deletions or duplications, leading to loss or gain of function. Therefore, mutations can guide changes in the biotransformation of drugs.^19^, ^20^, ^21^ The main genes belonging to this large family are *GSTM1* (μ), *GSTP1* (π), and *GSTT1* (θ).^22^

This study aims to determine the frequency of deletions and duplications in *CYP450* and *GST* genes in AML patients and their correlation with the neutrophilic recovery time (NRT) after remission induction treatment.

## Material and methods

The present study evaluated the genetic profile of the CYP450 and GST genes. Eighty-three DNA samples were included in the study, but thirteen were excluded because they were inadequate for multiplex ligation-dependent probe amplification (MLPA) analysis. Genomic DNA was extracted from leukocytes using the QiaAmp DNA Blood Mini kit (Qiagen-Germany) following the manufacturer's instructions.

The quality of the DNA was monitored by a NanoDrop Spectrophotometer (Thermo Fisher Scientific, USA), and the working concentration of the DNA was adjusted to 10∼50 ng/µL.

This study met all established ethical principles. All patients participating in the study signed informed consent forms. DNA samples were used where the patient died, as approved by the Research Ethics Committee (CAAE 48,471,221.3.0000.0068).

### Multiplex ligation dependent probe amplification (MLPA)

MLPA analysis was performed using the SALSA Probemix P128 CYP450 assay kit (MRC Holland, Netherlands) containing 52 MLPA probes with amplification products between 128 and 504 nucleotides. At least two probes are present for each of the target genes. Twelve-reference probes are included to detect autosomal chromosomal locations. The probes detect deletions or duplications in the *GSTM1, CYP1B1, CYP3A4, CYP3A5, CYP2C19, CYP2C9, CYP2E1, GSTP1, CYP1A2, CYP1A1, CYP2A6, CYP2B6, GSTT1* and *CYP2D6* genes.

Briefly, 250 ng of genomic DNA was hybridized to the probes following the manufacturer's protocol. Amplification was performed using the universal primers provided in the kit in a Veriti® Thermal Cycler (Applied Biosystems, USA). The cycling conditions were 30 s at 95 °C, 30 s at 60 °C, and 60 s at 72 °C for 35 cycles. For each run, three DNA samples were added from healthy individuals who did not present polymorphisms in the genes of interest according to previous analysis. Therefore, these samples were used as a control (reference) for comparative analyses. The polymerase chain reaction products were separated by capillary electrophoresis in the ABI 3130 automatic sequencer (Applied Biosystems, Foster City, USA). The peaks were analyzed using GeneMapper Software v4.0 (Applied Biosystems, USA), and data were analyzed with the Coffalizer Software (MRC Holland, Netherlands). This software provides quality indexes for each reaction, and normalizes the MLPA data by comparing each sample with a set of control samples. Patients with polymorphisms were considered when the final proportion (FP) between reference probes and sample were FP = 0 (homozygous deletion), 0.40 < FP < 0.65 (heterozygous deletion), 1.30 < FP < 1.65 (duplication in heterozygosity), and 1.75 < FP < 2.15 (duplication in homozygosity). Samples that had 0.80 < FP < 1.20 were normal.

### Analysis of neutrophil toxicity

Neutrophil toxicity was analyzed in 46 patients (65.7%). Minor neutrophil toxicity was characterized by a recovery time of up to 21 days to achieve ≥1.0 × 10^9^/L neutrophils, while major toxicity takes longer than 21 days to recover. The recovery time of 21 days was chosen because it allows for the start of a new cycle of chemotherapy in most protocols for the treatment of AML. It is important to closely monitor neutrophil toxicity and take appropriate action to prevent major toxicity, which can significantly impact treatment outcomes.

### Statistical analysis

The DNA samples extracted from patients with AML were done for convenience. Preliminarily, a descriptive statistical analysis of the genomic profile of deletions and duplications of the *CYP450* and *GST* genes was performed in patients diagnosed with AML. These findings are significant as they provide crucial insights into the genetic variations associated with AML. Categorical variables were analyzed for absolute and relative frequencies, while quantitative variables were assessed for median, interquartile range, and minimum and maximum values. Kaplan-Meier curves were constructed to compare the NRT of patients undergoing remission induction treatment and overall survival (OS). The differences in survival were estimated using the Kaplan-Meier method, and the differences between them and the cumulative rates of NRT were calculated using log-rank tests. The curves were compared using the non-parametric log-rank test, with a significance level of α = 0.05.

Additionally, Fisher's exact test assessed the correlation between TNR and AML risk classifications based on cytogenetic and molecular biology. It determined if patients with unfavorable risk had a distinct NRT compared to those with favorable risk (α = 0.05). The statistical analyses described above were performed using the statistical software R (version 4.3.0; https://www.r-project.org/) and RStudio (version 2022.12.0; https://www.rstudio.com/).

## Results

### Patient characteristics and polymorphisms presented

Table 1, Table 2 present the demographic data of the patients who participated in the study and the percentage of patients who presented or did not present polymorphisms according to self-declared ethnicity.

**Table 1:** Demographic characteristics of 70 patients.

Characteristic		
Age (years)		57 (19–81)
		n (%)
Sex	Female	40 (57.2%)
	Male	30 (42.8%)
Ethnicity	White	43 (61.4%)
	Brown	19 (27.1%)
	Black	5 (7.1%)
	Not declare	3 (4.3%)

**Table 2:** Polymorphism according to self-declared ethnicity.

	Polymorphisms	
Ethnicity	Present (*n* = 54)	Absent (*n* = 16)
		
White	32 (59.3%)	11 (68.8%)
Brown	18 (33.3%)	1 (6.3%)
Black	2 (3.7%)	3 (18.6%)
Not Declared	2 (3.7%)	1 (6.3%)

Table 3 shows the frequency of polymorphisms observed in patients according to the type (deletion and duplication) and the affected gene family (*CYP450* and *GST*). A striking observation is that 76 occurrences of polymorphisms were verified in the 54 patients, with some patients presenting polymorphisms in more than one family of genes. Consistent duplications in *CYP450* and *GST*, deletions in *CYP450* and *GST*, deletions in *CYP450* and duplications in *GST*, and duplications in *CYP450* and deletions in *GST* were observed (Supplemental material Figures 1, 2 & 3). The following genes were analyzed: *GSTP1, CYP1A1, CYP1A2, CYP2B6, CYP2C9*, and *CYP2C19*. However, none of the patients had any polymorphisms in these genes.

**Table 3:** Type of genetic aberration according to the affected gene.

Polymorphisms (*n* = 76)			
	***CYP450***	***GST***	**Total**
Deletion	14	24	38
Duplication	20	18	38
**Total**	34	42	76

### Neutrophil recovery time

In Table 4, the urgency of this research is underlined as toxicity levels are presented according to the NRT concerning the presence or absence of polymorphisms. It is alarming to note that patients who presented polymorphisms had a median TNR higher than patients who did not.

**Table 4:** Neutrophil recovery time (NRT) according to the presence or absence of polymorphisms.

	Polymorphisms (*n* = 46)	
NRT	Presence	Absence
NRT ≤21 days	0	6
NRT >21 days	20	20

The robust Fisher's exact test we applied to evaluate the NRT between groups with and without polymorphisms obtaining a p-value = 0.0287. This statistical significance further strengthens the research findings, instilling confidence in their validity.

**Figure 1:**
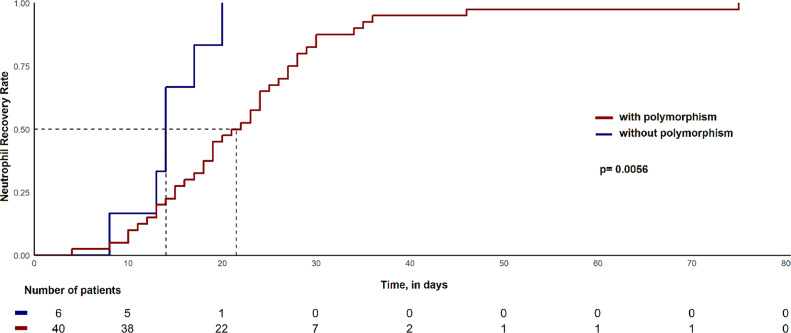
Estimated incidence of neutrophil recovery time between the group presented or not polymorphisms in *CYP450* and *GST.*

Figure 1 shows the cumulative rate of NRT. The median NRT for patients with polymorphisms of *CYP450* and *GST* was 21 days, while the median NRT for the group without polymorphisms was 12 days (*p*-value = 0.0056).

**Figure 2:**
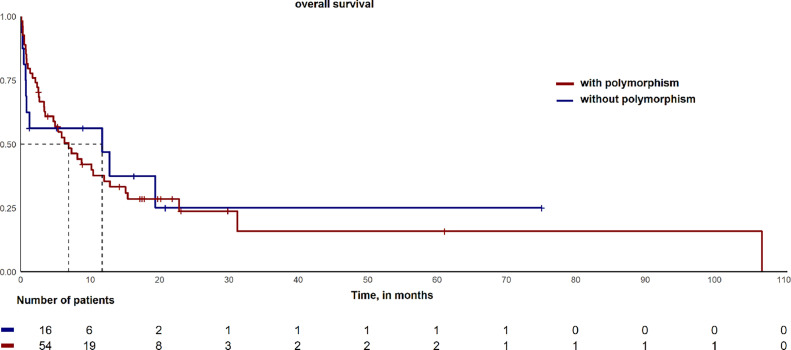
Comparison of probabilities of Overall Survival between the groups with and without polymorphisms.

Overall, the 9-year OS rate was assessed for 70 patients with a median follow-up time of 7.3 months. The estimated probabilities of OS were calculated and compared between patients with polymorphisms and those without. However, these two groups had no significant difference in the OS as shown in Figure 2.

## Discussion

AML is a severe condition for which the standard treatment is cytotoxic chemotherapy. However, genetic factors may affect the metabolism of these drugs, leading to prolonged hematological toxicity. Immediate treatment by healthcare professionals is necessary to avoid life-threatening complications.^23^^,^^24^

Previous studies have revealed that 43% of AML patients have a high mortality rate due to disease complications before they receive treatment. Studies have indicated that AML is more prevalent among white individuals and females The median age of patients is around 57 years.^25^ It is essential to note that the ethnicities reported in the present study were self-reported by patients and gathered from medical records. This may lead to some information bias in the results. The prevalence in the present study of genetic abnormalities in the *CYP450* and *GST* genes between different ethnicities, namely white, brown, and black, yielded similar results to those published in the literature.^26^^,^^27^ However, the white population had higher rates of *CYP450* and *GST* gene abnormalities (59.3%). Multiple genetic changes were found in the same gene family, an aspect that has also been reported previously in Brazilian population studies.^28^ The *CYP2D6* gene had the highest percentage of polymorphisms among the genetic alterations in the *CYP450* family (47%), and the prevalence of deletion-type polymorphisms in *GST* genes was evident, which aligns with the findings of previous studies.^28^

Assessing the neutrophil count is crucial to evaluate the potential hematological toxicity following chemotherapy. A low count of neutrophils (≤1.0 × 10^9^/L) could indicate this toxicity. The time neutrophilic cells take to recover after chemotherapy can vary depending on the type of chemotherapy regimen. Consequently, this study investigated the effect of polymorphisms in CYP450 and GST on the NRT, defined as 21 days. In this study, the NRT could be determined effectively in only 65.7% of the 70 patients. Among these, 87% (40/46) had polymorphisms of the CYP450 and GST genes. The remaining 13% did not present any polymorphisms. Interestingly, the NRT was ≤21 days for these six patients. On the other hand, for patients with polymorphisms, 50% had NRTss equal to or <21 days, while the other 50% had NRTs greater than 21 days (Fisher exact test: p-value = 0.028). Specific polymorphisms of *CYP450* and *GST* may be related to drug metabolism, confirming published information. The patients who lacked genetic alterations in the *CYP450* and *GST* genes achieved neutrophil recovery faster than those with polymorphisms (Figure 1: *p*-value = 0.0056).

The OS rate of adult patients diagnosed with AML can vary based on various factors like the socioeconomic and cultural profiles, genetic mutations, and access to treatment. Additionally, neutrophilic toxicity during treatment can affect OS since it leads to immunosuppression and makes the patient more susceptible to bacterial, fungal, and viral infections. In this study, we found no significant statistical difference when comparing the OS rates of patients with and without any polymorphisms, as shown in Figure 2.

During this study, we faced some limitations that need to be addressed. Firstly, the series was limited due to the significant number of patients who were diagnosed with AML but died before starting induction treatment. One of the preponderant factors was the delay in reaching a reference center for diagnosis. Secondly, the MLPA technique used requires a good quality and quantity of DNA, free from contamination or degradation. Lastly, it is essential to note that MLPA does not detect point mutations or analyze the entire coding region of genes.

## Conclusion

There is a need for more pharmacogenomic studies in Brazil, especially in the area of oncology and oncohematology. These studies can help understand how polymorphisms in genes related to drug metabolism can lead to medication toxicity in patients with these pathologies. AML is an emergency in terms of diagnosis and treatment due to its high mortality rate. This can cause significant myelotoxicity if the patient has a polymorphism in one of the genes responsible for drug biotransformation. In conclusion, pharmacogenomics is a crucial factor in pharmacotherapy and should be considered.

## Conflicts of interest

The authors declare no conflicts of interest.

## Appendix Supplementary materials

Image, application 1
