# Supplementary material for: Assessing the genetic profile of cytochrome P450 and glutathione S-transferases of patients diagnosed with acute myeloid leukemia
Source: Hematol Transfus Cell Ther. 2025 May 7;47(2):103759. doi: 10.1016/j.htct.2025.103759 (PMC12131003; doi:10.1016/j.htct.2025.103759)
Supplement: Supplementary file 1 [file mmc1.pdf]

Assessing the genetic profile of cytochrome P450 and glutathione S-transferases of patients diagnosed with acute myeloid leukemia

Supplementary Material

In Figures 1-4, examples of Multiplex Ligation-dependent Probe Amplification (MLPA) analysis results are shown regarding the detection of polymorphisms in patients

Figure 1 exemplifies the MLPA analysis result of Patient A, where no polymorphisms are observed. Figures 2, 3, and 4 show polymorphism results in three patients (B, C, and D).

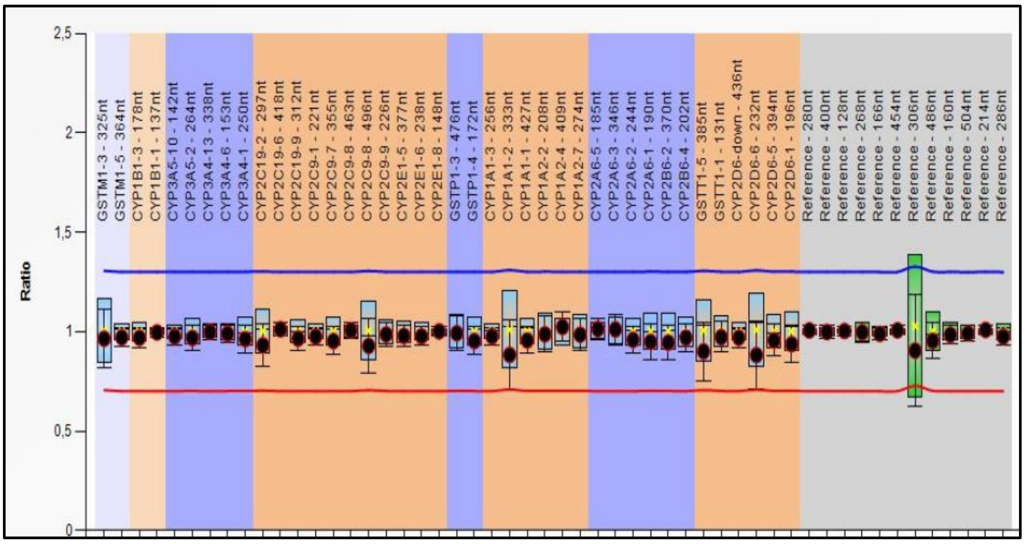

Figure 1: MLPA analysis result of Patient A showing absence of polymorphisms

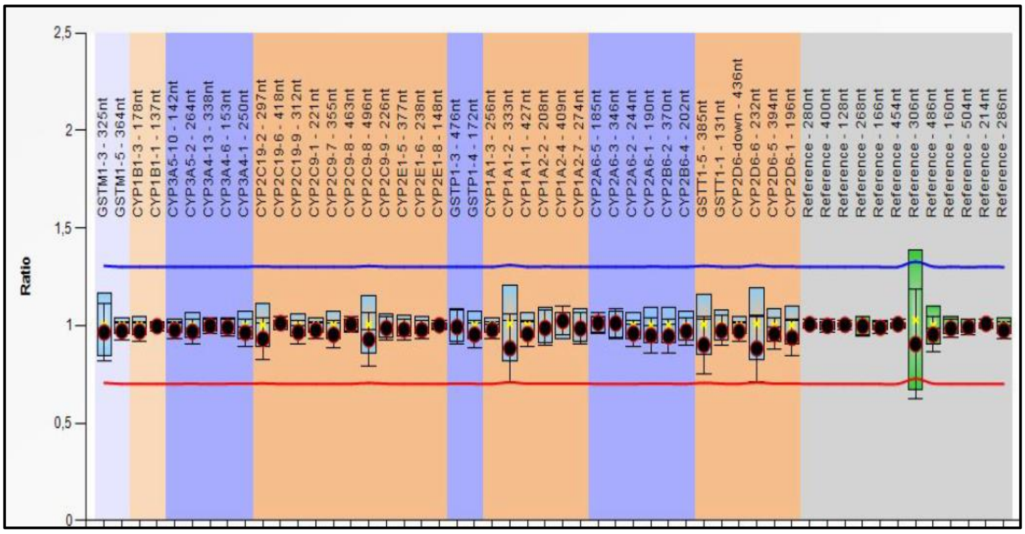

Figure 2: Duplication of exons 1 and 5 of the *GSTT1* gene and exon 1 of the *CYP2D6* gene in Patient B

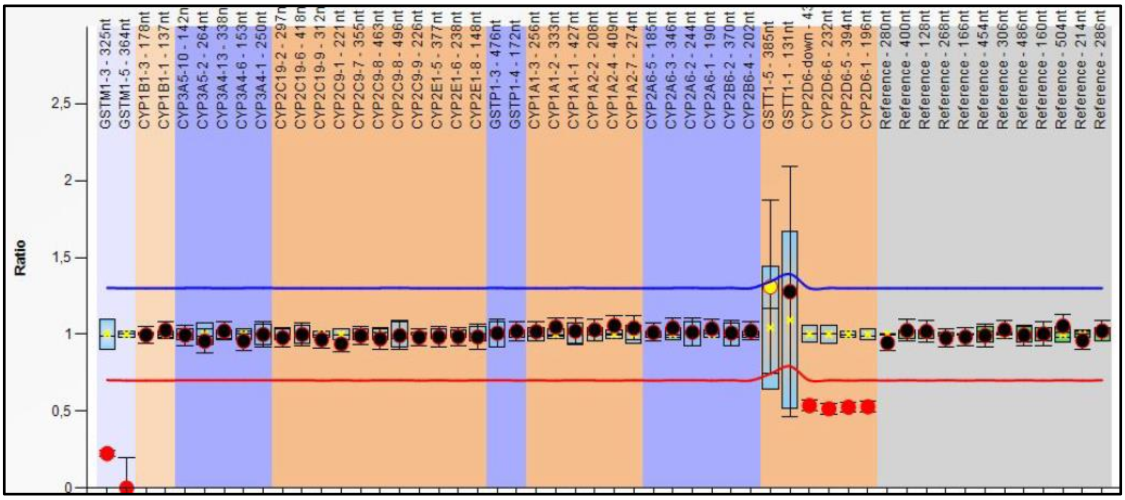

Figure 3: Deletions of exons 3 and 5 of the *GSTM1* gene and exons 1, 5, and 6 of the *CYP2D6* gene in Patient C

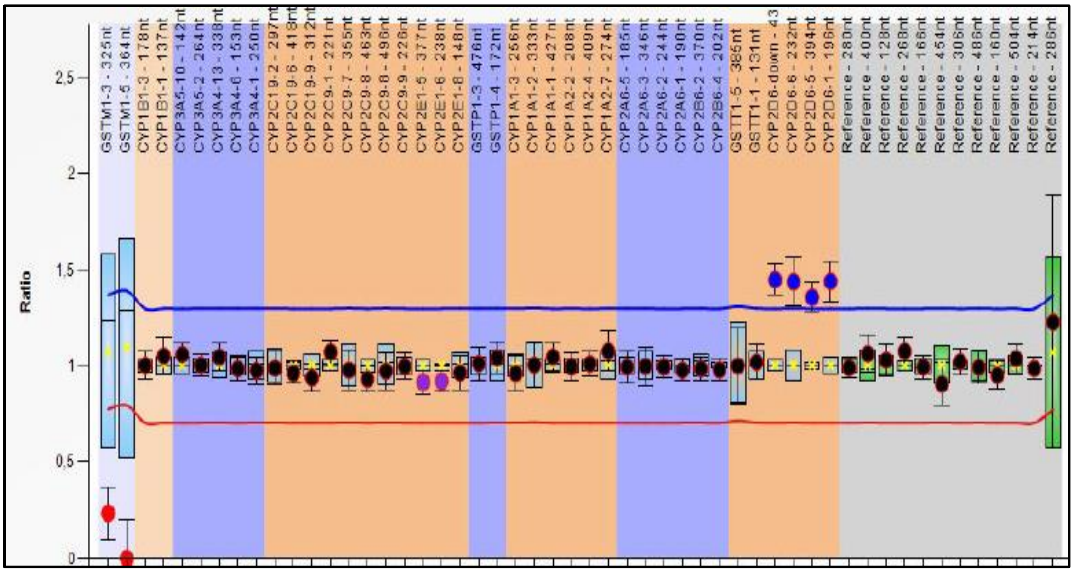

Figure 4: Deletion of exons 3 and 5 of the *GSTM1* gene and duplication of exons 1, 5, and 6 of the *CYP2D6* gene in Patient D
